# Application of Sunflower Husk Pellet as a Reducer in Metallurgical Processes

**DOI:** 10.3390/ma16206790

**Published:** 2023-10-20

**Authors:** Tomasz Matula, Jerzy Labaj, Pavol Vadasz, Beatrice Plešingerová, Albert Smalcerz, Leszek Blacha

**Affiliations:** 1 Department of Metallurgy and Recycling, Faculty of Materials Science, Silesian University of Technology, Krasinskiego 8, 40-019 Katowice, Poland; tomasz.matula@polsl.pl (T.M.); leszek.blacha@polsl.pl (L.B.); 2 Department of Production Engineering, Faculty of Materials Science, Silesian University of Technology, Krasinskiego 8, 40-019 Katowice, Poland; jerzy.labaj@polsl.pl; 3 Department of Non-Metallic Materials, Institute of Metallurgy, Technical University of Košice, Letna 9, 042 00 Kosice, Slovakia; pavol.vadasz@tuke.sk (P.V.); beatrice.plesingerova@tuke.sk (B.P.); 4 Department of Industrial Informatics, Faculty of Materials Science, Silesian University of Technology, Krasinskiego 8, 40-019 Katowice, Poland

**Keywords:** recycling of metalliferous raw materials, biomass, sunflower husk pellet, metallurgical process, copper

## Abstract

In relation to the climate policy being introduced, the search for a replacement for solid fossil fuels with renewable raw materials is ongoing. In this study, a potential biomass (sunflower husk pellet) application in the process of copper slag reduction was assessed. For the purpose of raw material characterisation, thermogravimetric tests were carried out and characteristic temperature points were determined with the use of a high-temperature microscope. The slag reduction tests led to the recovery of 97% of copper and a decrease in this metal content in the slag to less than 0.5% Cu, which enables safe storage or use in other industrial branches.

## 1. Introduction

Metal production is one of the basic factors affecting the economic development of the world. All existing technologies used for metal production using both primary and secondary raw materials yield, in addition to the basic product, i.e., metal, wastes that are eliminated during the particular production stage from the stream of materials being processed. For pyrometallurgical processes, such waste is slag, which is a very important element in these processes as it enables their proper course. For instance, it is a collector of contaminants released during the process.

In most cases, metallurgical slags should be utilised in accordance with the applicable environmental regulations. Considering the fact that the metal content in them is frequently higher than in primary raw materials, it is advisable to use this waste as the secondary metalliferous raw material. This method of slag utilisation is rational both for economic reasons and in the circular economy. It should be noted, however, that slags are metallurgical systems of complex structure and chemical composition, which determine the reactions of the reduction in the metal compounds contained in them [1,2]. In the case of slags derived from the technologies of copper production, this chemical element may appear as Cu+ cations or copper oxide. Considering the copper extraction from this type of material, the process is based on the course of a reduction reaction where a solid carboniferous reducer is mainly used [3,4]. It is assumed that the secondary slag generated in the analysed reduction process should contain less than 0.5 wt% Cu to be a safe material that can be stored or utilised in other branches. In this paper, the results of research on a potential use of biomass, i.e., sunflower husk pellet, in processes of copper slag reduction are presented. The concept of applying this type of biomass in the discussed process results from a meaningful decrease in coal mining and a limited production of coke and coke breeze observed in the EU countries in recent years [5]. These two raw materials and anthracite are the basic carboniferous materials utilised in the pyrometallurgical processes of metal production using both primary and secondary raw materials [6,7].

**Figure 1:**
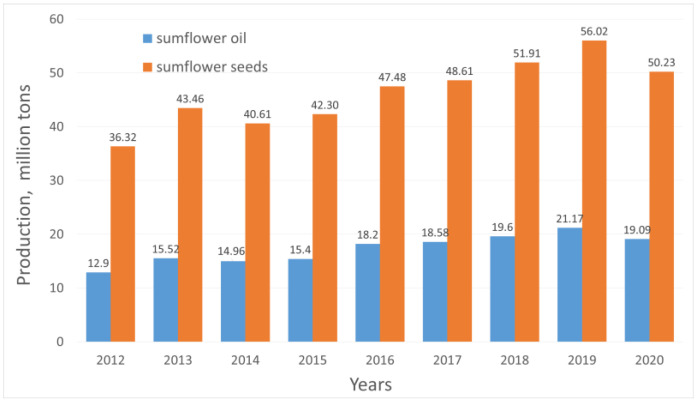
World production of sunflower seeds and sunflower oil.

The interest in husks from oil plant seeds as waste, with great potential to be applied as an alternate solid biomass fuel, has primarily focused on sunflower, peanut or coconut [8]. This results from the activities conducted in many European countries that are aimed at the gradual withdrawal of traditional fossil fuels, such as coal or brown coal, due to the current climate policy [9]. The current utilisation of biomass for energy purposes refers to the production of solid fuels and liquid biofuels [10]. The previously mentioned husks of oil plant seeds are directly used for the production of solid fuels in the form of pellets or pellet components with other kinds of biomass [11]. In recent years, the world scale of the production of sunflower seeds and sunflower oil has been estimated at levels of more than 50 million tons yearly and 22 million tons. In both cases, production growth has been observed during the recent decade. (Figure 1) [12,13]. The leading countries in this production are Ukraine, Russia, the EU countries and China. Assuming that sunflower husk constitutes 50% to 60% of the seed mass, the estimated amounts of waste, i.e., husk, are several million tons yearly. Initially, sunflower was grown only as an ornamental plant. Since the 17th century, sunflower seeds have been added to a variety of foods, or they have replaced coffee following thermal processing. It was not until the 19th century that oil was pressed from sunflower seeds. Sunflower is grown as a vegetable with edible seeds. It is one of the most popular oil plants [14]. Sunflower oil is extracted from its seeds and used in the food industry (e.g., to produce margarine) and as industrial fat used in the paint industry to produce varnish or lacquers [15].

The obtained laboratory test results indicate the possibility of using sunflower husk pellets in the process of metal recovery from metallurgical slags and thus partially replacing the standard coke breeze reducer. However, at this stage, the laboratory scale of the tests and the representativeness of the material used preclude the estimation of the costs associated with introducing sunflower pellets into the industrial process. For the purposes of this work, unit prices of coke breeze, sunflower husk pellets, and current ETS fees resulting from CO_2_ emissions were compared.

Based on the data from the literature (U.S. Energy Information Administration (EIA)) concerning the estimated consumption of coke in the copper production process and the prices of coke and carbonaceous material on the world markets, the average price for the analysis in 2022 was 107,66 USD/Mg of coke breeze, and it was assumed that the annual coke consumption in this process was at the level of 20,000 Mg. In this case, the unit cost of ETS permits per emission of one ton of CO_2_ should also be taken into account. In the reduction process using coal/coke, the unit cost is 80.70 EUR/ton [16].

Depending on the region in Europe, the cost of purchasing 1 ton of sunflower husk pellets ranges from 90 to 350 USD /ton. Offers from Russia and Belarus are much cheaper, but due to the restrictions imposed on these countries resulting from the aggression against Ukraine, they are unavailable.

The values of the share of elemental carbon per unit of slag indicate that in the case of the addition of biomass, a similar efficiency in the reduction process of approximately 50% is observed in relation to the coke breeze used. However, due to the content of volatile substances in the biomass, the content of which decreases significantly in the range of up to 400 °C, for technological reasons, it cannot completely replace a standard reducer. Determining the share of elemental carbon, due to process indicators, requires further research and an increase in scale.

Shelled sunflower seeds are rich in methionine and cysteine, which make them valuable components of the human diet [17,18]. Regarding sunflower husk pellet, its main technical application is associated with household and industrial energy [19,20]. The economic aspect, i.e., the cost of sunflower husk pellet production, is relatively low compared to the costs related to coal mining or the extraction of oil and natural gas [21]. Also, it should be emphasised that sunflower pellet is an environmentally friendly fuel [22]. It is assumed that the amounts of CO_2_ released during its combustion are equal to those observed during the natural decomposition of biomass [23]. In addition, this type of raw material is used in the production of, e.g., electricity, biofuels or polymer biocomposites [24]. In addition to high combustion heat values (compared to other biomass types), the advantages of employing sunflower pellet as an alternate fuel are low humidity and low contents of sulphur [25,26]. Another area of this biomass usage is gardening (for mulching), as sunflower husks demonstrate allelopathic properties [27,28]. Due to its high protein and vitamin contents, sunflower husk is also considered to be one of the most valuable and relatively cheap fodders for farm animals. [29]. Moreover, studies on the potential for using sunflower husk ashes in the ceramic industry and in the process of epoxy composite production have been conducted [30].

The available literature data related to the application of various biomasses in high-temperature processes of metal production show that this kind of research referred to their potential use in processes of metal ore oxide reduction [31,32], metal recovery from secondary raw materials [33,34,35], or iron ore sintering [36,37,38,39,40]. In addition, advanced studies on the application of various biofuels in metallurgical processes are being conducted. In the case of sunflower husk pellet, the available data are limited and only refer to its use as a carboniferous material in the process of iron ore sintering [41,42,43].

## 2. The Present Study

The following research programme was developed to determine the potential for use of sunflower husk pellet in the process of metallurgical slag reduction:

1. Determination of copper slag melting temperature;
2. Sunflower pellet degasification tests using the thermogravimetric method;
3. Copper slag reduction tests using sunflower pellet.

### 2.1. Research Materials

A reducer in the form of sunflower husk pellet and copper slag were the tested materials. Their chemical compositions are presented in Table 1.

Based on the literature data [44,45,46,47,48], a list of the chemical compositions of sunflower husk pellets from various areas of the world was prepared (Table 2).

**Figure 2:**
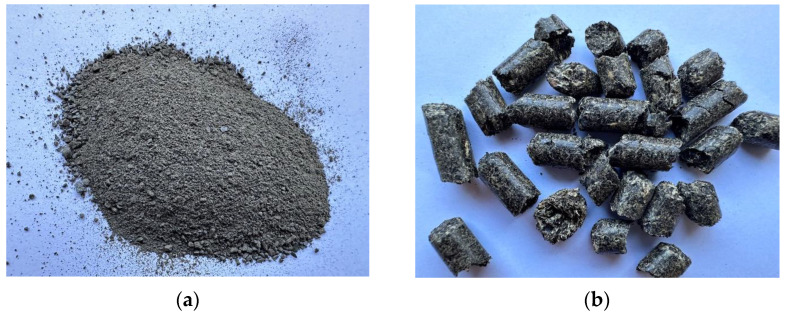
Images of the tested (**a**) is slag (**b**) is pellet.

A preliminary study of the microstructure and the phase composition of the slag demonstrated its complex morphology and significant differences in particle sizes from several hundred nanometres to several micrometres. The identified particles were characterised by clearly specified surfaces: flat, rounded, and spherical. The results of the phase analysis of the slag showed the presence of iron and copper, mainly in the oxide form, i.e., Fe_3_O_4_, Fe_2_O_3_, and Cu_2_O [49]. The images of the tested slag and pellet are presented in Figure 2.

### 2.2. Equipment and Research Methods

The method of determining the fusibility of metallurgical slags is based on the evaluation of shape changes for the tested body (powdery sample pressed to a cylinder/cube) during its continuous heating in the set atmosphere (oxidising or reducing). The temperatures at which characteristic changes occur are recorded (image or video). According to the international standard, the defined fusibility temperatures are determined by the characteristic shapes of a deformed body during heating, such as [50]:

(a) Deformation temperature (DT)—the first signs of the sample edge rounding define the start of material softening;
(b) Spherical shape temperature (ST)—the softening temperature is defined by the shape of the sample with completely rounded edges (sphere), without a change in its height;
(c) Hemispherical shape temperature (HT)—the melting temperature is defined approximately by the hemispherical shape of the sample, where the height is equal to the half of the diameter of the base;
(d) Flow temperature (FT)—the temperature is defined by a flowing sample with 1/3 of the height of the tested sample at the melting temperature (HT).

**Figure 3:**
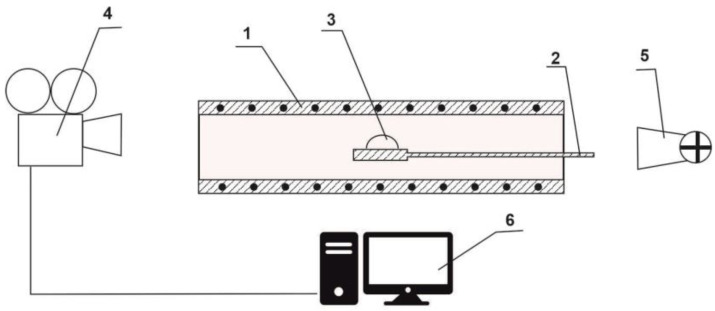
Diagram of a high-temperature microscope: 1—high-temperature furnace, 2—substrate, 3—sample, 4—camera, 5—source of light, 6—computer. Crushed samples were homogenised/pulverised in a porcelain dish to a grain size below 0.063 mm. The powder sample was pressed without any addition of binder in a hand press (2.7 MPa) to form a cylindrical tablet (φ 3 mm × 3 mm).

The test of the metallurgical slag’s fusibility was carried out using a Leitz-Wetzlar high-temperature microscope with a Kanthal Super furnace at temperatures up to 1500 °C with heating rates of 10 ± 1 °C.min^−1^. The sample was placed in a holder (corundum tube) on a corundum plate. The temperature value was scanned with a Pt-PtRh10 thermocouple (Figure 3). The measurements were carried out in a static oxidising (air) atmosphere. The shape profile of the tested sample was scanned constantly using a digital camera (Progressive Scan IP) with an optical grid (mesh = 0.5 mm × 0.5 mm) with a magnification of 10× and transferred onto a PC with a recording and on a screen (photo or video) [51].

During the measurement, the temperature and the shape of the tested sample were recorded on photographs or video recordings. Each record of the sample shape included sample identification, the corresponding temperature, and the date/time of the measurement. An allowed tolerance of temperature deviation is ±10 °C [50]. On the basis of these records, a report of sample fusibility was completed, including sample identification, the temperature of deformation, spherical shape, hemispherical shape, flow temperature, reference to the used standard method (measuring procedure), and essential measurement parameters.

The thermogravimetric tests were carried out using an STA 449 F3 (Jupiter, Netzsch, Selb, Germany) thermal analyser. The device contained a graphite furnace operating in the protective environment in the presence of a selected gas or gas mixture. Before each experiment, a slag sample and the reducer of a precisely defined weight were placed inside a small DTA/TG Al_2_O_3_ crucible, which was then attached to the measuring head in the working chamber of the analyser. The following parameters were recorded during the tests: sample mass loss, temperature, and test duration. Before the experiment, a sunflower pellet sample of a particular weight (approx. 200 mg) was placed inside a small DTA/TG Al_2_O_3_ crucible, which was then attached to the measuring head in the working chamber of the analyser. The measurements were performed in an argon atmosphere with hydrogen addition. The assumed programme of sample heating consisted of three essential stages. The first stage involved sample heating up to 1200 °C (20 °C/min); next, the sample was isothermally held for 30 min; finally, it was cooled down to 800 °C. The amount of volatile matter in the tested material corresponded to the loss of sample mass recorded during its heating as part of the experiment.

The tests of reduction melting of copper slag were carried out in a Czylok PT 40/1300 (Jastrzebie, Poland) laboratory resistance furnace. Its diagram is presented in [52]. The use of this type of furnace during the laboratory tests enabled easy charge loading and process control. The reduction process was carried out in alundum crucibles at 1300 °C. This temperature was selected based on the findings of the slag fusibility tests. The weight of the slag sample undergoing the reduction tests was 100 g. The variable parameters in the tests were:

- The reducer (pellet) weights: 5.67 g and 7.56 g, respectively;
- Duration of the reduction process (1 h to 4 h).

The weight of smelted alloy was determined after each experiment. In addition, the metal and slag yielded during the reduction process underwent a chemical analysis for the copper content. The analyses were carried out using a Rigaku Primus II X-ray fluorescence spectrometer.

## 3. Test Results and Discussion

### 3.1. Determination of the Copper Slag Melting Temperature

**Figure 4:**
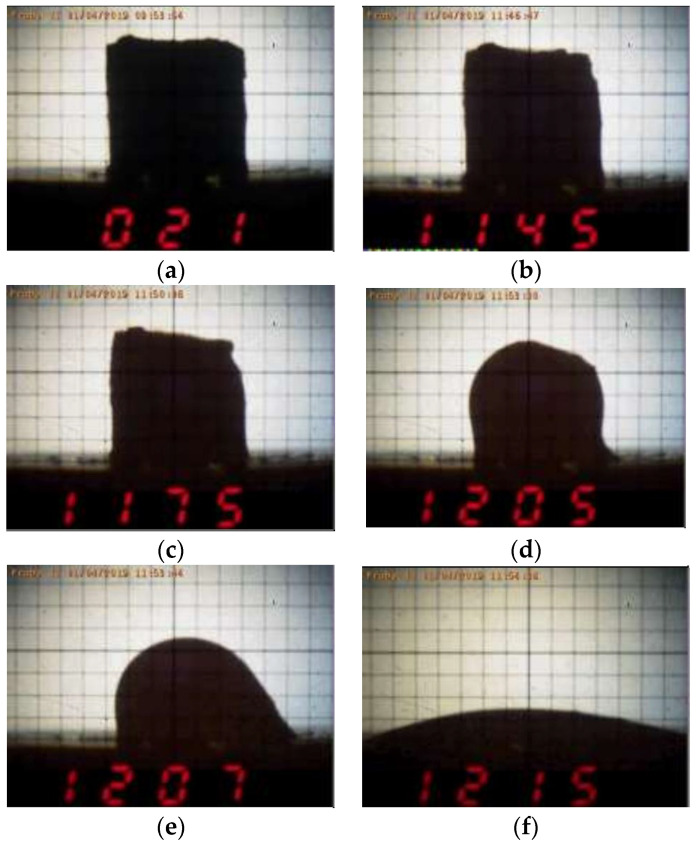
Images of the slag sample during its heating up at (**a**) 21 °C, (**b**) 1145 °C, (**c**) 1175 °C, (**d**) 1205 °C, (**e**) 1207 °C, (**f**) 1215 °C.

Based on the tests carried out using a high-temperature microscope, the determined values of the characteristic temperatures of the tested copper slag are presented in Table 3. In this case, the set temperature in the process of slag reduction was 1300 °C. Examples of the slag sample images during its heating up to 1350 °C are presented in Figure 4.

### 3.2. Tests of Sunflower Pellet Degasification

**Figure 5:**
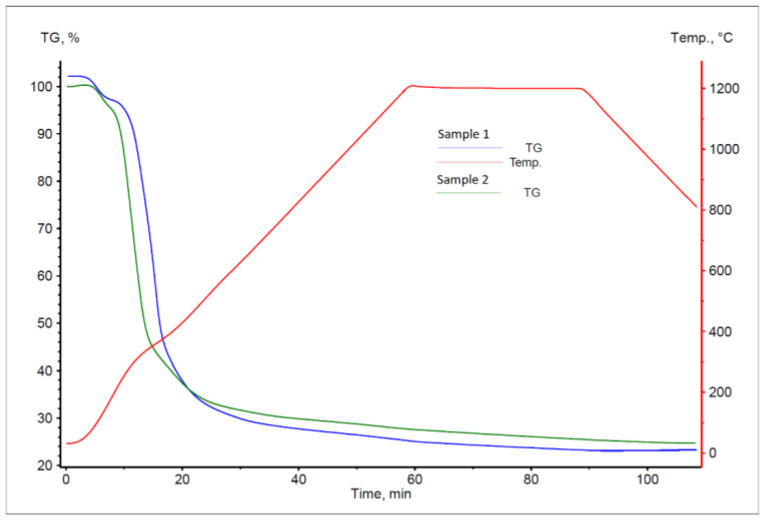
The TG curve for the process of sunflower pellet sample degasification.

**Figure 6:**
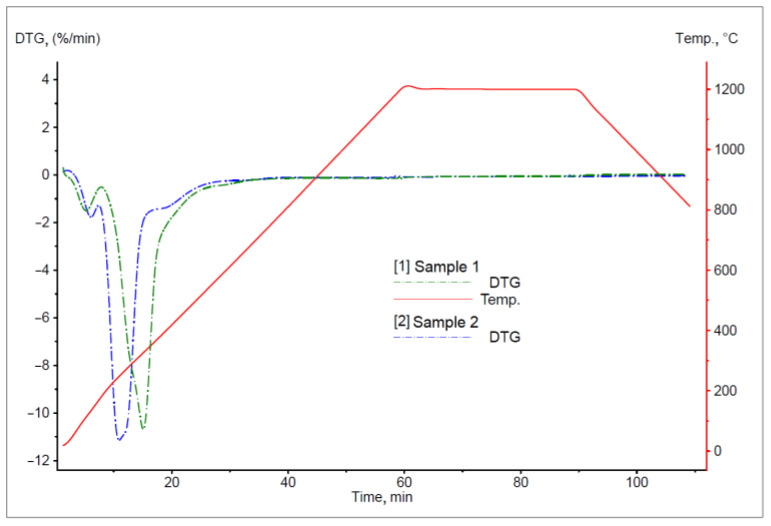
The DTG curves for the process of pellet sample degasification.

The TG curves for the degasification process for the sunflower pellet samples are presented in Figure 5. The DTG curves for these samples are presented in Figure 6.

The analysis of the results of thermogravimetric tests showed that sunflower pellet contained over 75% volatile matter. The shapes of the plotted TG curves indicate three distinct temperature ranges, which significantly differ in the aspect of mass loss (Table 4). The first mass loss recorded for both samples was associated with the evaporation of moisture, and the second one related to the evaporation of oil substances contained within sunflower husks. The latter process occurs at temperatures that are not MUCH higher than 200 °C. The next decrease in the sample mass was associated with the decomposition of hemicellulose (250–300 °C), cellulose (300–350 °C), and lignin (above 400 °C) [53].

**Figure 7:**
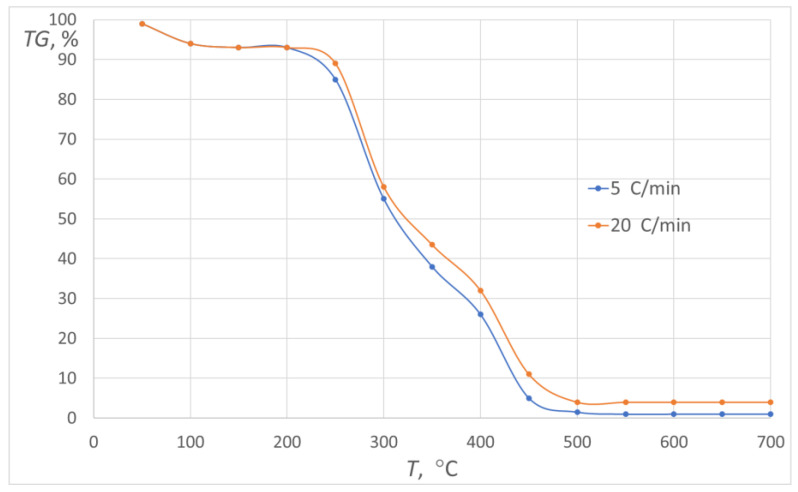
The TG curves for sunflower pellet for the tests in the oxidising atmosphere.

It should be noted that methane can be released during the process of sunflower pellet sample heating in an inert atmosphere [54]. This gas is an additional factor with reducing properties in the analysed process of slag reduction. For comparison, when the process of sunflower pellet sample heating occurs in an oxidising atmosphere, a greater sample mass loss is observed (over 95%), which mainly results from carbon and hydrogen combustion [55,56]. Examples of the TG curves for the process of sunflower pellet heating in an oxidising atmosphere at various heating rates are presented in Figure 7 [57].

### 3.3. Slag Reduction Tests

The results of the copper slag reduction process with the use of sunflower husk pellet as the reducer are presented in Table 4. In addition to the basic process parameters, the table contains data referring to the amounts of metal and secondary slag after the reduction process, as well as the contents of copper in the secondary slag and in the metal.

The performance indicator for the studied process of copper slag reduction was the decopperisation degree *S_Cu_*. It was determined based on the following equation:

(1) $$S_{Cu}=\frac{\left(C_{Cu}^{0}–C_{Cu}^{k}\right)}{C_{Cu}^{0}}\cdot 100\%$$

where $C_{Cu}^{0}\;\mathrm{and}{\;C}_{Cu}^{k}$ represent the initial concentration of copper in the slag (wt%) and the final concentration of copper after the process of slag decopperisation (wt%), respectively.

The values of this indicator and the comparative results for the slag reduction with the use of coke breeze (reference material) are presented in Table 5.

**Figure 8:**
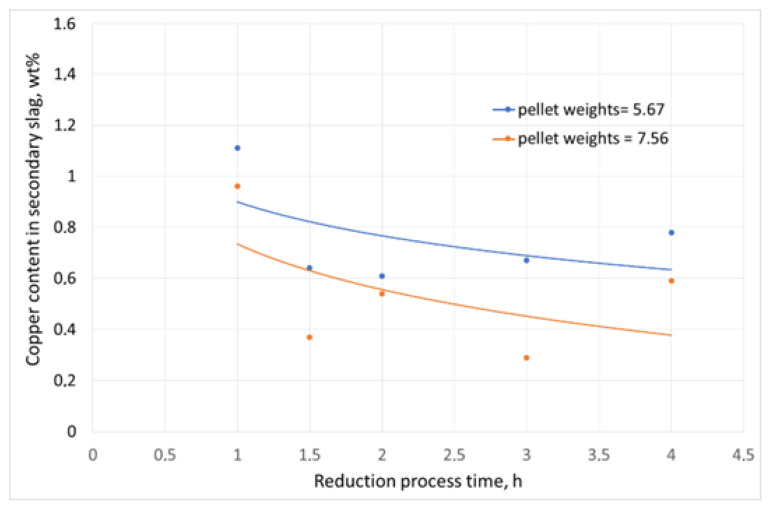
Copper content in secondary slag after the reduction process.

**Figure 9:**
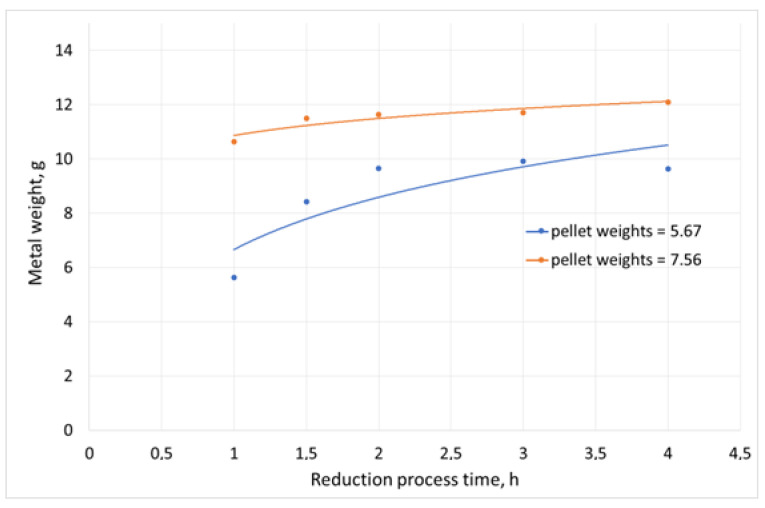
Metal weight after the slag reduction process.

**Figure 10:**
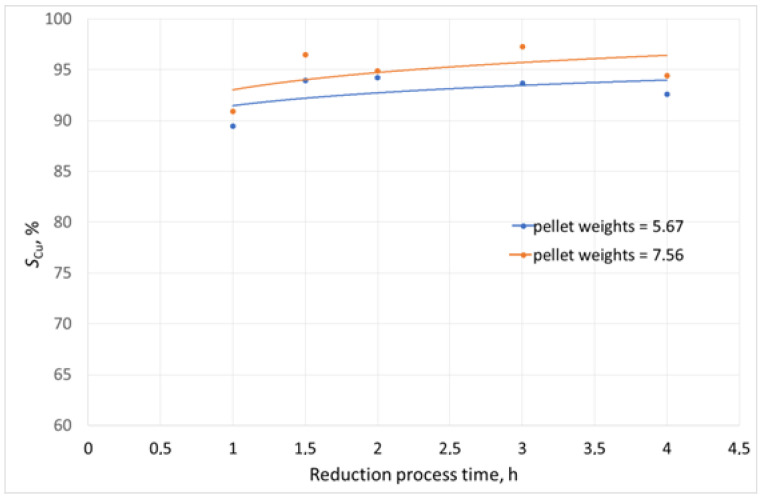
Slag decopperisation degree.

The graphical interpretations of the results are presented in Figure 8, Figure 9 and Figure 10.

While analysing the results of changes in the copper content in the slag after the reduction process, its decrease from 10.3 wt% (the content in the initial slag) to less than 1.2 wt% could be observed in each experiment. Moreover, the degree of slag decopperisation ranged from 89% to 97%. For comparison, in the experiments where coke breeze was used, the decrease in copper content in the slag after the process from 5.25 wt% to 0.67 wt% was found, which corresponded to the degree of decopperisation, which ranged from 50.1% to 93.63%. When the reducer addition was increased and the duration of the reduction process was extended, greater weights of the smelted metal phase were observed. Smaller contents of copper were seen in the smelted alloy when the reduction duration was extended due to higher amounts of lead in the alloy, as a result of the reduction of the metal oxide compounds in the slag. Also, it should be noted that the reduction of lead and iron compounds contained in copper slags follows the reduction of copper compounds, which was demonstrated experimentally [56].

The laboratory tests of copper slag reduction with the use of sunflower husk pellet confirmed the potential for the recovery of copper and its accompanying metals. Sunflower husk pellet showed a high content of volatile matter, including moisture, oil substances, and methane, which may have a positive effect on the course of the reduction process due to the better blending of reagents. To assess the efficiency of the process, the degree of decopperisation was determined, which was 89.46% for the process carried out at 1300 °C for 1 h, with 5.67% of the reducer addition up to 97.25% for the process carried out for 3 h and with 7.56% of the reducer addition. Further extension of the process duration did not affect the value of the decopperisation degree. The conducted tests of the slag reduction process with the use of coke additive, i.e., a standard reducer in the metallurgical process, in the amount of 5.76 g allowed to achieve a copper removal rate of 89.72% Cu—93.63% Cu. Considering the parameter “Amounts of carbon per 1 g of slag” (Table 4), it can be seen that almost twice as many units of carbon per unit of slag were required to achieve a comparable degree of copper separation compared to sunflower husk pellets. In each reduction test, the addition of 5.67% of sunflower husk pellet led to a decrease in the copper content in secondary slag from 1.11% Cu to 0.61% Cu, while the addition of 7.56% of sunflower husk pellet resulted in a decreased copper content in secondary slag from 0.96% Cu to 0.29% Cu. From a technological point of view, the acceptable copper content in secondary copper slag should not exceed 0.5% Cu. In addition to a comparable efficiency of the process, the industrial application of sunflower husk pellet as a coke breeze substitute (the standard reducer) will be beneficial due to a limited carbon footprint, because the amount of CO_2_ released during the reduction process is the same as that released during the natural process of biomass decomposition.

## 4. Conclusions

Based on the experiments, the following conclusions were formulated: The presented experimental results showed the possibility of using biomass in the form of sunflower pellets as a reducer in the process of copper recovery from copper slag. Using this type of reducer, a decopperisation rate of 89 to 97% was achieved. The values of the decopperisation degree were higher than those obtained in the reduction tests conducted using coke, which is a typical reducer used in the discussed technological process. An additional advantage of using sunflower pellets as a coke replacement is the neutrality in terms of carbon dioxide emissions.

## Figures and Tables

**Table 1:** Chemical compositions of sunflower pellet and copper slag.

**Composition of the tested sunflower pellet**					
Pellet component	C	H	O	N	S
Component content, wt%	48.0	5.92	49.1	0.52	0.09
**Composition of the tested copper slag**					
Slag component	Cu	Pb	Fe	SiO_2_	CaO
Component content, wt%	10.3	2.25	11.1	34.5	14.1

**Table 2:** Chemical compositions of sunflower pellet (literature values).

Composition of the Tested Sunflower Pellet					
Pellet Component	C	H	O	N	S
Component content, wt%	42.1–69.8	5.17–8.8	30.36–48.92	0.33–1.15	0.09–0.24

**Table 3:** Characteristic temperatures of the tested copper slag.

Sintering Temperature	From 1145 °C
Deformation temperature	1175 °C
Softening temperature	1205 °C
Melting temperature	1207 °C
Flow temperature	1215 °C

**Table 4:** Recorded mass losses of the particular samples.

	Sample 1			Sample 2		
Sample mass loss	Loss I, %	Loss II, %	Loss III, %	Loss I, %	Loss II, %	Loss III, %
	4.47	68.25	5.54	4.38	65.33	5.67
Temperature range for the particular loss, °C	20–230	230–890	890–1200	20–180	180–760	760–1200
Mass loss rate, mg	0.0090	0.0346	0.0021	0.0088	0.0391	0.0019

**Table 5:** Results of slag copper removal experiments.

No	Type of Reducer	Amounts of the Reducer, g	Reduction Time, h	Amounts of Carbon per 1 g of Slag, g	Metal Weight, g	Secondary Slag Weight, G	Copper Content in the Metal, wt%	Copper Content in Secondary Slag, wt%	*S_Cu_*, %,
1	Sunflower husk pellet	5.67	1	0.0284	5.64	89.13	91.00	1.11	89.46
2		7.56		0.0378	10.64	84.01	88.70	0.96	90.88
3		5.67	1.5	0.0284	8.42	85.97	94.90	0.64	93.92
4		7.56		0.0378	11.49	85.08	88.30	0.37	96.49
5		5.67	2	0.0284	9.65	86.81	85.40	0.61	94.21
6		7.56		0.0378	11.64	85.69	92.50	0.54	94.87
7		5.67	3	0.0284	9.92	88.41	88.41	0.67	93.64
8		7.56		0.0378	11.71	87.01	86.80	0.29	97.25
9		5.67	4	0.0284	9.63	87.66	86.20	0.78	92.59
10		7.56		0.0378	12.09	86.53	86.30	0.59	94.40
11		10	3	0.0500	12.26	85.41	85.66	0.41	96.11
12		12		0.0600	13.26	84.1	88.08	0.5	95.25
13	Coke breeze	5.42	1	0.0700	2.05	95.25	92.10	5.25	50.10
14			2		3.50	92.13	93.23	1.01	90.41
15			3		6.70	87.36	90.13	1.08	89.72
16			4		8.41	83.95	87.58	0.67	93.63

## Data Availability

Data sharing is not applicable to this article.
